# CDinFusion – Submission-Ready, On-Line Integration of Sequence and Contextual Data

**DOI:** 10.1371/journal.pone.0024797

**Published:** 2011-09-13

**Authors:** Wolfgang Hankeln, Norma Johanna Wendel, Jan Gerken, Jost Waldmann, Pier Luigi Buttigieg, Ivaylo Kostadinov, Renzo Kottmann, Pelin Yilmaz, Frank Oliver Glöckner

**Affiliations:** 1 Max Planck Institute for Marine Microbiology, Bremen, Germany; 2 Jacobs University gGmbH, Bremen, Germany; 3 Fachhochschule Bingen, Bingen am Rhein, Germany; Baylor College of Medicine, United States of America

## Abstract

State of the art (DNA) sequencing methods applied in “Omics” studies grant insight into the ‘blueprints’ of organisms from all domains of life. Sequencing is carried out around the globe and the data is submitted to the public repositories of the International Nucleotide Sequence Database Collaboration. However, the context in which these studies are conducted often gets lost, because experimental data, as well as information about the environment are rarely submitted along with the sequence data. If these contextual or metadata are missing, key opportunities of comparison and analysis across studies and habitats are hampered or even impossible. To address this problem, the Genomic Standards Consortium (GSC) promotes checklists and standards to better describe our sequence data collection and to promote the capturing, exchange and integration of sequence data with contextual data. In a recent community effort the GSC has developed a series of recommendations for contextual data that should be submitted along with sequence data. To support the scientific community to significantly enhance the quality and quantity of contextual data in the public sequence data repositories, specialized software tools are needed. In this work we present CDinFusion, a web-based tool to integrate contextual and sequence data in (Multi)FASTA format prior to submission. The tool is open source and available under the Lesser GNU Public License 3. A public installation is hosted and maintained at the Max Planck Institute for Marine Microbiology at http://www.megx.net/cdinfusion. The tool may also be installed locally using the open source code available at http://code.google.com/p/cdinfusion.

## Introduction

The introduction of the first deoxyribonucleic acid (DNA) sequencing methods in 1977 marked a major breakthrough in life science [1], [2]. Subsequently, developments in these technologies allow the routine sequencing of organismal genomes, metagenomes and marker genes from all domains of life. Genomic information can be seen as the ‘blueprint’ of life and being able to decode and to interpret it, grants insight into life's fundamental mechanisms [3], [4]. However, microbes pose a challenge to genomic description as the vast majority of microbial life cannot readily be isolated in pure cultures [5], [6]. The rise of cultivation independent approaches like metagenomic and sequencing of marker genes addresses this limitation [7]. In these approaches, bulk DNA is extracted from an environmental sample and either specific genes are amplified and sequenced or random sequencing is performed. Thus, a fragmented, but cultivation-independent, overview of an environment's biological diversity and functional potential is provided [8], [9].

Early on, scientists recognized the necessity to share sequence data to facilitate reuse, reproducibility and comparisons. This has become an integral part of the research and publication process. In the ‘Bermuda Principles’, on the first international strategy meeting on human genome sequencing in 1996, it was agreed upon, that all human genomic sequence information, generated by centers funded for large-scale human sequencing, should be freely available in the public domain to encourage research and to maximize its benefits to society (http://www.ornl.gov/sci/techresources/HumanGenome/research/bermuda.shtml, accessed:11.03.2011). In the Fort Lauderdale meeting in 2003 organized by the Wellcome Trust, it was finally agreed to deposit all kinds of sequencing data that are analyzed in scientific publications in public databases. Over the past two decades, the amount of sequence data submitted to the world's largest public nucleotide sequence data repository INSDC (International Nucleotide Sequence Database Collaboration, comprising of DDBJ (DNA Data Bank of Japan), ENA (European Nucleotide Archive), and GenBank) has grown exponentially [10]. Recently, Next Generation Sequencing (NGS) technologies [11] allow even faster and more economical sequence generation, resulting in an unprecedented sequence accumulation.

Despite the impressive magnitude of sequence data generation, numerous life science studies have shown that contextual (meta)data (CD) are crucial for their interpretation [12]–[14]. CD are metadata about features such as the environmental origin and the processing steps that were applied to obtain the sequences. These range from data about the geographic location (latitude, longitude), sampling time, habitat, to experimental procedures used to obtain the sequences up to video data recorded during sampling. The fact however that e.g. latitude, longitude (INSDC: lat_lon), and time (INSDC: collection_date), which can be submitted to the public repositories for years, have so far only been reported in 7.3% and 7.2% of all submissions [15], strongly implies that the procedure to deposit these data is hampered. Common reasons are: 1) no clear descriptors exist to guide the submitters which metadata should be deposited and 2) no appropriate tools exist that support the combined submission of sequence data and CD.

These concerns have recently prompted the Genomic Standards Consortium (GSC), an international consortium, which promotes mechanisms to standardize the description of genomes and the exchange of genomic data, to create a series of checklists defining the minimal set of CD that should accompany a sequence submission. The Minimum Information About a (Meta)Genome Sequence (MIGS/MIMS) checklist [16] outlines a conceptual structure for extending the core information that has been traditionally captured by the INSDC (DDBJ/EMBL/GenBank) to describe genomic and metagenomic sequences. The Minimum Information about a MARKer gene Sequence (MIMARKS) standard complements the MIGS/MIMS specification by adding two new “report types”, a “MIMARKS-survey” and a “MIMARKS-specimen”, the former being the checklist for uncultured diversity marker gene surveys, the latter is designed for marker gene sequences obtained from any material identifiable via specimens. The standards also cover sets of measurements and observations describing particular habitats, termed “environmental packages”. Collectively the MIGS/MIMS/MIMARKS standards are now called MIxS (Minimum Information about any (x) Sequence) [17], [18]. Through collaboration with the GSC, the INSDC now offers the structures to store the data items specified in the GSC checklists. This facilitates an early integration of sequence data and CD. However, specialized tools to allow this integration for different user scenarios are needed.

The European Nucleotide Archive (ENA) provides an on-line submission system called Webin which contains prepared web forms for the submission of GSC compliant data. It shows all fields with descriptions, explanations and examples and does data validation in the forms (https://www.ebi.ac.uk/embl/genomes/submission/login.jsf, accessed: 16.03.2011). The Investigation Study Assay (ISA) Infrastructure offers a software suite that produces documents that can be submitted to the Sequence Read Archive (SRA) repository [19]. With the Quantitative Insights Into Microbial Ecology (QIIME) web application [20] users can generate and validate MIMARKS-compliant templates. Finally, MetaBar is a spreadsheet and web-based software tool which assists users in the consistent acquisition, electronic storage and submission of CD associated to their samples [15]. However, a tool that integrates CD and sequence data by directly enriching FASTA files for submission does not exist yet.

Here we present CDinFusion (Contextual Data and FASTA in fusion). CDinFusion has been designed to submit sequence data together with CD to INSDC. CDinFusion intends to facilitate the integration of CD and sequence data prior to submission by directly enriching sequence data using the FASTA format. It generates submission-ready outputs for INSDC by implementing the MIxS standard defined by the GSC. CDinFusion processes single as well as MultiFASTA files, containing up to millions of sequences. It was successfully applied to several use cases. Example submissions to the INSDC can be accessed with the following accession numbers: JF681370, JF268327–JF268425 and Genome Project ID 63253. A public installation is hosted and maintained at the Max Planck Institute for Marine Microbiology, Bremen, Germany: http://www.megx.net/cdinfusion. The tool is easy to install and released under the LGPL 3 open source license to promote distribution in aid of increasing the quantity and quality of CD in the public repositories.

## Results and Discussion

CDinFusion has been designed as a web-based tool, which enables users to upload single or MultiFASTA files from single sequence to high-throughput analysis and enrich them with CD. After uploading the sequences, the user is requested to select the appropriate GSC checklist and environmental package. CD can be entered in the web forms or CSV templates can be downloaded, filled with CD off-line and uploaded. The CSV files help to store and share the data. The merged sequence and CD can be downloaded for subsequent submission to INSDC.

**Figure 1:**
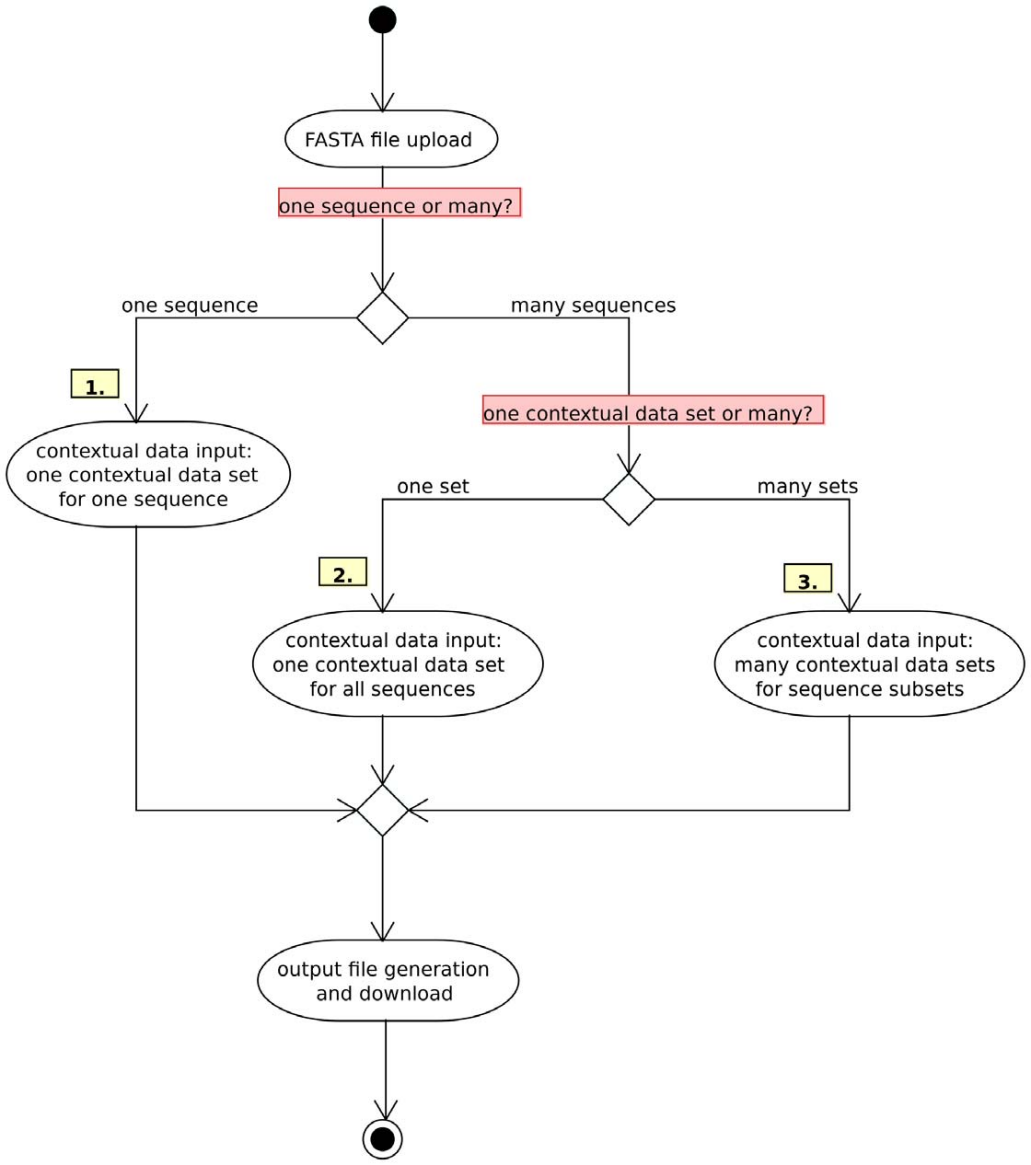
Overview of submission scenarios. Three primary scenarios of sequence data submission to INSDC can be distinguished and are all covered by the CDinFusion workflow:1) The submission of a single FASTA sequence file along with one CD set, 2) The submission of a MultiFASTA file along with one CD set for all sequences in the file and 3) The submission of a MultiFASTA file annotated with several CD sets.

The implemented workflow covers the three typical scenarios of sequence submission to an INSDC database namely: 1) Enriching a single sequence with one CD set, 2) Enriching many sequences in a MultiFASTA file with one CD set, and 3) Enriching subsets of sequences in a MultiFASTA file with several CD sets (Figure 1).

**Figure 2:**
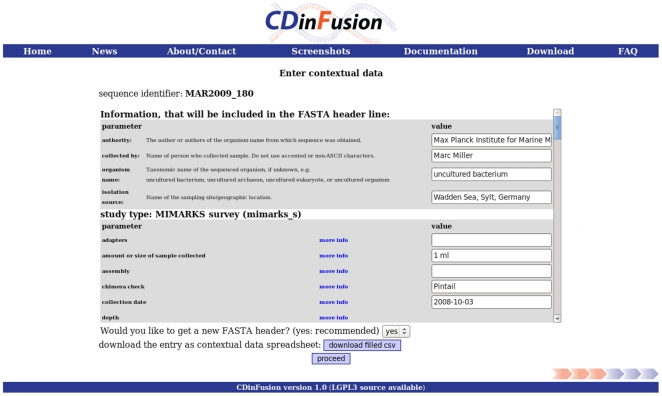
CDinFusion web user interface. The CD are entered into the auto-generated web forms. Details about each parameter are accessible with the “more info” link. These details are retrieved using a web service accessing the GSC database and are therefore always up to date.

The functionality of each of these different scenarios has been tested in dedicated use cases. The first use case was conducted with a single 16S rRNA sequence obtained from a bacterium isolated from a coastal water sample taken off the coast of the Wadden Sea island Sylt. After uploading the FASTA file the tool directly proceeded to the CD package selection for one CD set, as the file contained only a single sequence. The MIMARKS survey (mimarks_s) package and the water package were selected to provide suitable CD fields for this environmental survey sequence obtained from seawater. Subsequently the web forms were filled with all the CD available for this particular sequence (example Figure 2). After generating and downloading the output file, the CD enriched FASTA was imported into Sequin version 11.00. CDinFusion inserted qualifiers specified by GenBank into the header line of the FASTA file. The tool placed the rest of the CD into a tab delimited structured comment file. This file was loaded into Sequin with the “Advanced Table Readers” option in the “Annotate” menu. The CD appeared in the metadata section between the header and the feature table section. By selecting “Done”, the Sequin file was saved and the complete submission was prepared. The INSDC database entry for this submission can be accessed at [Accession number: JF681370].

This use case exemplifies submission scenarios, where a single sequence and its CD are to be submitted to the INSDC databases. Single sequences can, for example, be marker genes or genomes that consist of a single sequence or contig.

In the second use case, a permanent draft genome from a *Rhodopirellula baltica* strain along with its associated CD was prepared for submission. After the 6.9 Mb MultiFASTA file was uploaded, the user was offered the option to annotate all sequences in this file with one CD set or to enter many CD sets for sequence subsets. As all sequence fragments were parts of the same bacterial genome, isolated from a sediment sample, one CD set for all sequences was selected using the MIGS bacterial genome (ba) checklist and the sediment package. The user filled in all CD fields available and the CD enriched files were generated, downloaded and imported into Sequin. The data of this genome project can be accessed by ID 63253 and with the accession number: AFAR00000000. The genome will be analyzed in a separate study in preparation (Richter et al., Permanent draft genome sequence of *Rhodopirellula baltica* WH47).

This use case describes a procedure that may also be applied to metagenomic MultiFASTA files originating from one sampling site, which should be annotated with the same CD.

In the third use case a MultiFASTA file containing 99 16S rRNA sequences, obtained from a clone library, was enriched with CD. This file comprised four sequence subgroups, each with distinct CD. After the MultiFASTA file was uploaded, the CD for each of the groups was entered sequentially until all sequence subgroups were annotated. After the user selected the MIMARKS (mimarks_s) and the “environmental package” sediment the CD were entered in the web forms.

The output files created were a CD enriched MultiFASTA file and a compressed ZIP archive containing four structured comment files, one for each of the subgroups. After the FASTA file had been imported to Sequin, the structured comment files were loaded one by one with the “Advanced Table Readers” function. The file was then saved and submitted. This clone library and its CD [21] will be analyzed in a separate study in preparation (Ruff et al., Microbial Communities of Submarine Methane Seeps at Hikurangi Margin, New Zealand). The INSDC database entries for this submission will be available under the accession numbers: JF268327–JF268425.

The same procedure has been applied to ten 16S rRNA sequences of an environmental culturability study conducted by the M.Sc. Marine Microbiology (MarMic) class of 2014 at the island of Sylt. The sequences of that study will be analyzed in a separate study in preparation (Hahnke et al., *Flavobacteria* of the North Sea: Diversity of Culturability) available under the accession numbers: JF710778–JF710788.

This use cases apply, whenever batches of sequences have to be submitted and subgroups of these sequences have to be annotated with individual CD sets. These MultiFASTA files can for example contain batches of marker genes or a pooled metagenome.

To test if high-throughput data can be processed with CDinFusion, metagenomic FASTA files from the Global Ocean Survey (GOS, http://jcvi.org/cms/research/projects/gos/overview/, accessed: 16.03.2011), and metagenome data from the Microbial Interactions in Marine Systems project (MIMAS, http://www.mimas-projekt.de/mimas/, accessed: 16.03.2011) were loaded into CDinFusion. FASTA files containing over two million sequences with file sizes of two GigaBytes (GB) could be processed in less than three minutes in an AMD™ 64Bit, 2 GHz and 4 GB RAM environment.

All described test cases were recorded with the Selenium IDE (http://seleniumhq.org/) test case recorder. The test cases along with the test data, except for the metagenomic datasets, are deposited at http://code.google.com/p/cdinfusion. Descriptions how to run the tests, can be found in the documentation section of the public CDinFusion installation at http://www.megx.net/cdinfusion.

## Materials and Methods

### Languages, Tools and detailed Workflow

CDinFusion has been designed to allow users to add CD to single and MultiFASTA files that may comprise one to several million sequences. The CD enriched output can readily be submitted to the INSDC archives. The tool is programmed in the object-oriented, platform-independent programming language Java SE 5.0 (http://www.oracle.com/technetwork/java/index.html) using the Eclipse IDE (http://www.eclipse.org/). The open source Spring framework (http://www.spring-source.org/about/) was used, which supports the Model-View-Controller (MVC) design pattern. The functionality of the tool was continuously tested using the Selenium IDE (http://seleniumhq.org/). It runs on an Apache Tomcat 5.5.25 web server (http://tomcat.apache.org/). The project has been built using Apache Ant 1.7.1 (http://ant.apache.org/) and has been deployed on a web server with 2 GHz AMD Opteron™ processor 246, with 4 GB main memory and Debian GNU/Linux 5.0.3 (lenny).

**Figure 3:**
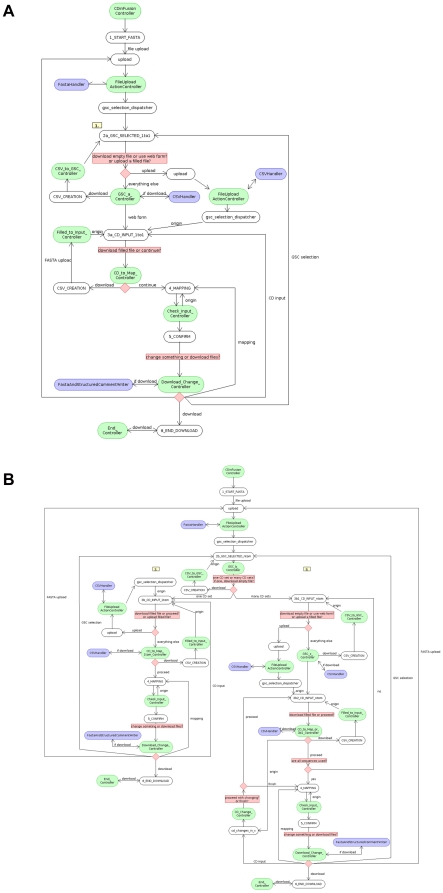
CDinFusion implementation details. The implementation details along the workflows 1–3 covering the primary scenarios of sequence data submission to the INSDC are shown. CDinFusion implements the Model-View-Controller design pattern. Classes implementing the data model and its manipulation methods are shown in blue, components belonging to the web user interface (view) are shown in white and components directing the workflow (control) are shown in green.

Figures 3a and 3b show the implementation details of the software's workflow. FASTA files are parsed and validated, when uploaded by the FastaReader class. It implements the FastaValidatorCallback interface of the FastaValidator package (http://www.megx.net/FastaValidator), which has been developed within the frame of this project. This event-driven parser is designed to quickly parse and validate arbitrarily large FASTA files with minimal time and memory requirements. It facilitates the processing of gigabases of FASTA files containing millions of sequences on common desktop PC architectures. The parser is available separately and is also released under the GNU LGPL 3 license. It may also be used for other projects.

If only one sequence is detected in the FASTA upload, the control flow will be directed towards the 2a_GSC_SELECTED_1to1 JSP (use case 1 in the Results section), shown in Figure 3a. If the user opts to annotate all sequences of a MultiFASTA file (Figure 3b) with either one CD set or many CD sets, the control flow will be directed either to the 3b_CD_INPUT_1tom JSP (use case 2 in the Results section) or to the 3b1_CD_INPUT_ntom JSP (use case 3 in the Results section), respectively.

After the CD have been entered into the web forms, these data may be downloaded as comma separated value (CSV) files. The CSV files may serve as local backups and can be edited off-line and uploaded to CDinFusion to re-populate the web forms. Each session concludes with a confirmation step, where users can revisit any previous step and correct CD input if necessary. This holds true for all three branches of the workflow (Figure 3a and 3b). If the user chooses to proceed to the file download, a CD FASTA file and a structured comment file are generated and can, depending on their size, either be imported to Sequin or merged on the command line using tbl2asn (http://www.ncbi.nlm.nih.gov/genbank/tbl2asn2.html, accessed: 30.03.2011) before submission.

### Implementation of the GSC checklists in CDinFusion

Once the user has uploaded a MultiFASTA file and its contents have been validated, the data is processed along the data model (Figure S1). For each CD set a CDElement is created that contains an object for a “type of report” and an object for an “environmental package”. The GSC MIxS standard, including all “type of reports” and the “environmental packages”, is maintained in a relational database system called the GSC database at the Max Planck Institute for Marine Microbiology Bremen on behalf of the GSC. A non- authoritative version of the database can be downloaded at http://gcdml.gensc.org/wiki/GscDb [17]. Java classes were auto-generated from the relations in the GSC database using the Ibator tool from the iBatis project (http://ibatis.apache.org). The Java classes cover the MIGS, MIMS and MIMARKS (MIxS) specifications. The GSC plans to refine these standards annually. With every new version of the standards the Java classes can easily be updated using the Ibator tool.

The short names of the parameters are resolved using a web service that was developed within the frame of this project. The web service offers details about all GSC parameters stored in the GSC database. Web forms (see Figure 2) are dynamically rendered during runtime and therefore always contain the latest information including all definitions and descriptions of the GSC checklists parameters. If a user wants to know how a certain GSC parameter is specified, the “more info” link opens a window with information about the full name of the parameter, its definition, the expected value, the syntax and an example. This information is directly retrieved from the GSC database. For CDinFusion to be fully functional, there needs to be Internet access to the web service. If a certain type of report and environmental package has been selected, these parameters are cached. The next time these packages are selected the web forms are rebuild from cache without re-using the web service.

Two Strings “first SequenceID” and “lastSequenceID” in the CDElement object store the range of the associated sequence identifiers for each CD set. The CDFastaHeader object contains those parameters that are covered by the web forms in addition to the GSC parameters that are later used to extend the FASTA header lines.

### Installation details

There are two ways to install CDinFusion: 1) CDinFusion can be installed by downloading and deploying the pre-compiled web archive file (war) on an Apache Tomcat (version >5.5.25). In this case the war file only has to be uploaded in the Tomcat manager. Afterwards the application can be accessed under http://<local_tomcat_installation>/CDinFusion. This method is preferable if users do not want to compile the program from its source code. 2) CDinFusion can also be installed by downloading and compiling the source code and subsequently deploying the software on an Apache Tomcat web server (version >5.5.25). To compile the code, the generic build.xml and build.properties files can be adjusted to local settings. If the standard settings in these files are not changed, the war file will be compiled into the CDinFusion root folder. The project can be compiled by executing the Apache ant build tasks, “deploy” or “deploywar”, respectively. The build.xml can additionally be configured to directly deploy the tool on an Apache Tomcat web server or to create the war file and upload it with the Tomcat manager. Further installation details can be found in the README.txt file that is included in the source bundle and that is also available in the documentation section of the CDinFusion web page. On some platforms the CATALINA_HOME environment variable needs to be set, in order for CDinFusion to write and read files. Relative to the path specified, CDinFusion will create a “data” folder, where temporary files will be saved. The application has been tested on Debian GNU Linux installations, but should be platform-independent and run on all platforms that support Java and Apache Tomcat installation such as Windows™ or MAC OS™.

### Availability and Future Directions

The public installation of CDinFusion is hosted and maintained at the Microbial Genomics and Bioinformatics Group (MGG) of the Max Planck Institute of Marine Microbiology Bremen and accessible under: http://www.megx.net/cdinfusion. The source code is available under GNU LGPL 3 and deposited in a public repository: http://code.google.com/p/cdinfusion.

As open source software it is the intention of the MGG to support this software well into the future. Currently CDinFusion supports submission of CD enriched sequence data to the INSDC using Sequin and tbl2asn for large data sets. Support for installations outside the MPI cannot be granted. The direct submission to EMBL/ENA and DDBJ is planned. Furthermore the integration of GCDML [22] as an exchange format would be advantageous. The GSC and life science community is encouraged to download the source code and to modify and extend the software to make it even more useful.

## Supporting Information

Figure S1 **In the CDinFusion data model the central Java class is the CDElement class, which is a composition of the classes “report type” and “environmental package”.** These classes implement the MIGS, MIMS and MIMARKS (MIxS) checklists specified by the GSC. The two strings “firstSequenceID” and “lastSequenceID” define if the CDElement contains CD for a single or a range of sequences. Instances of the CDFastaHeader class contain the data that is generated into the FASTA headers in the FASTA file.
(TIF)
