# Supplementary figures and images for: CDinFusion – Submission-Ready, On-Line Integration of Sequence and Contextual Data
Source: PLoS One. 2011 Sep 13;6(9):e24797. doi: 10.1371/journal.pone.0024797 (PMC3172294; doi:10.1371/journal.pone.0024797)

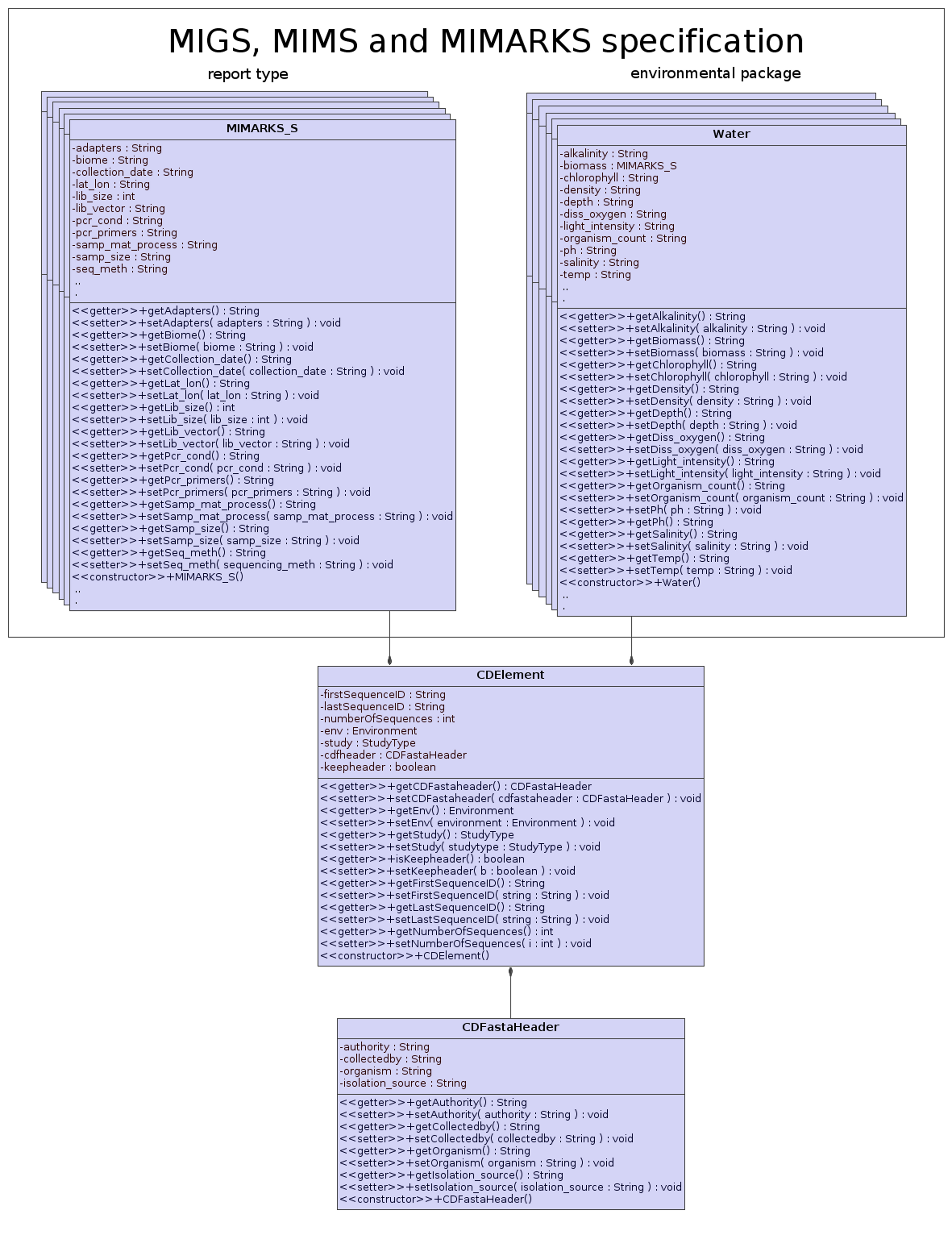

Supplement: Figure S1 — In the CDinFusion data model the central Java class is the CDElement class, which is a composition of the classes “report type” and “environmental package”. These classes implement the MIGS, MIMS and MIMARKS (MIxS) checklists specified by the GSC. The two strings “firstSequenceID” and “lastSequenceID” define if the CDElement contains CD for a single or a range of sequences. Instances of the CDFastaHeader class contain the data that is generated into the FASTA headers in the FASTA file. (TIF) [file pone.0024797.s001.tif]
